# Overexpression of hypoxia-inducible factor and metabolic pathways: possible targets of cancer

**DOI:** 10.1186/s13578-017-0190-2

**Published:** 2017-11-13

**Authors:** Davinder Singh, Rohit Arora, Pardeep Kaur, Balbir Singh, Rahul Mannan, Saroj Arora

**Affiliations:** 1 Department of Botanical and Environmental Sciences, Guru Nanak Dev University, Amritsar, 143005 India; 2 Department of Biochemistry, Sri Guru Ram Das University of Health Sciences, Amritsar, 143001 India; 3 Department of Pharmaceutical Sciences, Guru Nanak Dev University, Amritsar, 143005 India; 4 Department of Pathology, Sri Guru Ram Das University of Health Sciences, Amritsar, 143001 India

**Keywords:** mTOR signaling pathway, HIF-1α, Hypoxia, Metabolic pathways, p53, Glycolysis

## Abstract

Cancer, the main cause of human deaths in the modern world is a group of diseases. Anticancer drug discovery is a challenge for scientists because of involvement of multiple survival pathways of cancer cells. An extensive study on the regulation of each step of these pathways may help find a potential cancer target. Up-regulated HIF-1 expression and altered metabolic pathways are two classical characteristics of cancer. Oxygen-dependent (through pVHL, PHDs, calcium-mediated) and independent (through growth factor signaling pathway, mdm2 pathway, HSP90) regulation of HIF-1α leads to angiogenesis, metastasis, and cell survival. The two subunits of HIF-1 regulates in the same fashion through different mechanisms. HIF-1α translation upregulates via mammalian target of rapamycin and mitogen-activated protein kinase signaling pathways, whereas HIF-1β through calmodulin kinase. Further, the stabilized interactions of these two subunits are important for proper functioning. Also, metabolic pathways crucial for the formation of building blocks (pentose phosphate pathway) and energy generation (glycolysis, TCA cycle and catabolism of glutamine) are altered in cancer cells to protect them from oxidative stress and to meet the reduced oxygen and nutrient supply. Up-regulated anaerobic metabolism occurs through enhanced expression of hexokinase, phosphofructokinase, triosephosphate isomerase, glucose 6-phosphate dehydrogenase and down-regulation of aerobic metabolism via pyruvate dehydrogenase kinase and lactate dehydrogenase which compensate energy requirements along with high glucose intake. Controlled expression of these two pathways through their common intermediate may serve as potent cancer target in future.

## Background

Cancer is a known worldwide threat responsible for ~ 7.6 million deaths per year, which is expected to reach 13.1 million by 2030 [1]. Cancer, a multifactorial disease is the second main cause of human deaths after cardiovascular diseases. Biological systems have various pathways to suppress cancer propagation such as tumor suppresser genes, cell cycle check points, DNA error repair system etc. Down regulation or malfunctioning of these system results in initiation of cancer. Over expression of hypoxia inducible factor (HIF) and altered metabolic pathways are two classical features of cancer [2]. HIF-1 is a transcription factor regulating many pivotal pathways in normal as well as cancerous cells. It is over expressed in organs or tissues where oxygen level drops below threshold level [3]. High level of HIF-1 points towards angiogenesis, cell proliferation, survival and tumor progression through regulation of growth promoters, oncogenes, glycolytic pathways and pH regulation. A large number of studies support the relation of increased level of HIF-1 with aggressive tumor growth and poor patient prognosis [4–8]. Metabolic pathways are crucial for growth and survival of cells. Intensively proliferating cells (as in cancer) needs high rate of energy and thus metabolic pathways are modified to match the need. The anaerobic condition results in drastic drop of energy production as lower number of ATPs are produced. Up-regulation of glycolysis and regulated feedback systems solve this problem. These two factors (HIF-1 and metabolic pathways) help cancer cells in rapid proliferation and also for angiogenesis, metastasis and evading apoptosis. This review is thus compiled to analyze the role of HIF-1 and altered metabolic regulation in cancer.

## Hypoxia-inducible factor

### Structure

**Fig. 1:**
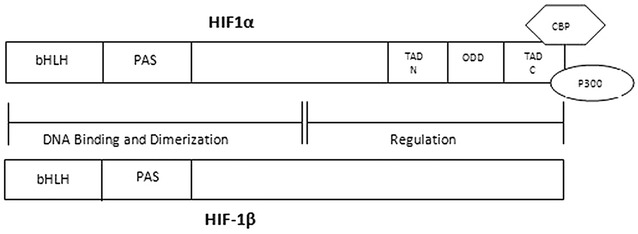
Structure of HIF-1α and HIF-1β gene. These genes contain a common basic helix-loop-helix and PAS domain for DNA binding and dimerization. HIF-1β lacks an N-TAD, C-TAD and ODD regulatory regions

HIF is a heterodimer protein consisting of two subunits, HIF-α and HIF-β. There are two other substitutes of HIF-1α such as HIF-2α and HIF-3α [9, 10]. Both HIF-1α and HIF-2α have the ability to heterodimerize to HIF-1β subunit because of 85% sequence similarity in bHLH domain [11]. Expression of HIF-α subunit is oxygen dependent while HIF-β constitutively expresses independent of oxygen level. HIF-β subunit is also known as aryl hydrocarbon receptor nuclear translocator (ARNT) and binds to aryl hydrocarbon receptor (AhR) to promote its translocation to the nucleus [12]. Both HIF-α and HIF-β subunits belong to bHLH-PAS (basic helix loop helix-Per ARNT Sim) protein family found in Drosophila [13]. All three HIF-α subunits contain oxygen-dependent degradation domain (ODD) and N-terminal transactivational domain (N-TAD). In addition, HIF-1α and HIF-2α also contain a C-terminal transactivational domain (C-TAD) [14], but HIF-1β lacks all regulatory regions (Fig. 1). The ODD domain is crucial for activity and stability of HIF-α subunits as it contains proline and asparagine for hydroxylation under normoxic conditions [15]. Some co-activators such as C-TAD binding protein (CBP) and P300 bind with C-TAD and regulate HIF expression by altering local chromatin structure through lysine acetyltransferase (KAT) activity and interaction with core transcriptional machinery [16].

### Functions

**Fig. 2:**
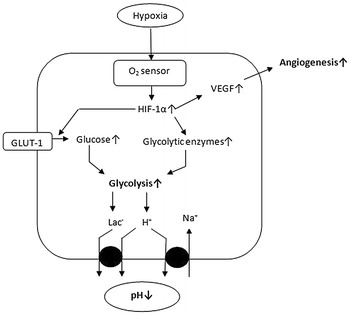
Major roles of HIF-1; regulation of angiogenesis through VEGF, pH through sodium hydrogen pumps and glycolysis through glycolytic enzymes as well as glucose transporters

Human tissues need ample supply of oxygen to maintain constant energy level through aerobic metabolic pathways. In some disorders such as cancer, chronic obstructive pulmonary disorders, ischemia or heart diseases, the oxygen level is drained, thus leading to hypoxic condition [17]. These conditions up-regulate HIF expression and down-regulate its degradation. HIF plays an important role in various cell signaling pathways besides three major roles, to regulate angiogenesis, metabolic pathways and pH (Fig. 2). Hypoxia is mainly common in solid tumors, where cells proliferate very fast, resulting in compression of blood vessels. To compensate this problem, HIF up-regulates the angiogenic growth factors such as vascular endothelial growth factor (VEGF) [18] and epidermal growth factor (EGF) through transcriptional activation. HIF enhances endothelial cell migration towards tissues with low oxygen level. These cells aid to form new blood vessels to overcome oxygen requirement [19]. VEGF enhances the infiltration of macrophages through macrophage receptors (flt-1). These macrophages release more VEGF and tumor necrosis factor- alpha (TNF-α) indicating positive regulation. HIF-1 also regulates glycolysis which is a vital pathway for energy production. It regulates the uptake of glucose through glucose transporters, i.e. glucose transporter 1 (GLUT1) and sodium–glucose transporters (SGLT) [20]. In absence of oxygen, metabolic pathways shift from more productive oxidative phosphorylation to less efficient anaerobic metabolism for maintenance of ATP production (Warburg effect) [21]. This shift is done through up-regulation of hexokinase, aldolase, pyruvate kinase and down-regulation of pyruvate dehydrogenase which promotes the conversion of pyruvate to acetyl CoA to enter the citric acid cycle [22]. Recently, it has been observed that the embryonic stem cells go through metabolic shift during development towards glycolysis which is regulated by HIF-1 [23].

Tumor cells have acidic extracellular pH (6.2–6.8) than normal cells (7.2–7.4) [24, 25]. More lactic acid production and poor removal, as well as the production of carbonic acid, are main reasons for this condition. Monocarboxylate transporters (MCT4) upregulates by hypoxic conditions, which secrete more lactic acid from tumor cells [26]. Also, HIF-induced ectoenzyme CA (carbonic anhydrase) IX or XII converts diffused carbon dioxide into carbonic acid to reduce extracellular pH [27]. Histochemical studies have shown that CA IX and CA XII isoforms expressed highly in tumor cells, which are thus a suggestive diagnostic marker for cancer.

## Regulation of HIF-1

**Fig. 3:**
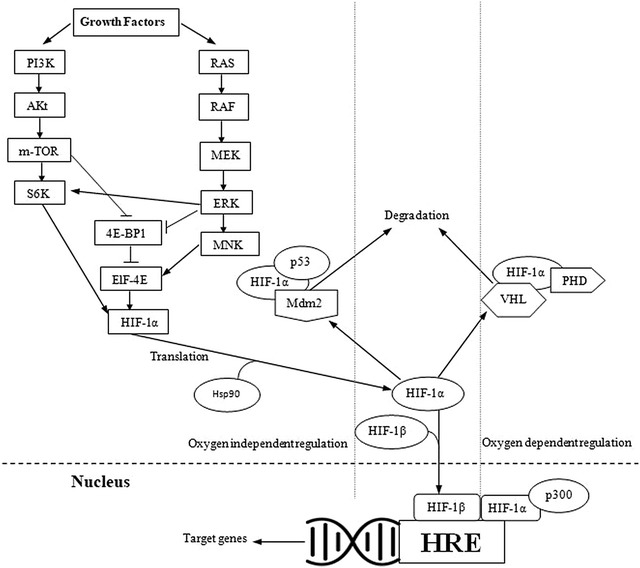
Oxygen-dependent and independent regulatory pathways of HIF-1

HIF-1α constitutively expresses independently of oxygen level through various signaling pathways in cancer [28]. Most post-translational modification accounts for HIF-1α stability and transcriptional activity via ubiquitination, acetylation, sumoylation, hydroxylation, and phosphorylation [28]. There are two types of the pathway which regulate the expression of HIF-1α, oxygen dependent and oxygen independent pathway (Fig. 3).

### Oxygen-dependent regulation

#### pVHL dependent pathway

In normoxic conditions, level of HIF-1 is tightly regulated by von Hippel-Lindau protein (pVHL), a tumor suppressor protein through proteasomal degradation and ubiquitination [29]. Another enzyme called prolyl-4-hydroxylases (PHDs) or HIF-1 prolyl hydroxylases (HPH) also participates in the degradation of HIF-1α. Two proline residues (P^402^/P^564^) present on KXXLAP amino acid motif of ODD domain acts as a substrate for PHDs [30, 31]. This degradation requires 2-oxoglutarate (2-OG), oxygen and ascorbate [32]. An enzyme, arrest defective-1 (ARD-1) plays an important role by acetylating lysine (K^532^) residue in the ODD domain [33]. Hypoxic condition inhibits the activity of both the enzymes resulting in up-regulation of HIF-1α activity. Hypoxia disrupts the electron transport chain, thus accumulating reactive oxygen species (ROS), which in turn oxidizes Fe^2+^ to Fe^3+^ resulting in inhibition of PHD activity and stabilization of HIF-1α. Knockdown of transferrin receptor-1 through small hairpin RNA (shRNA) leads to decrease in iron uptake, high HIF expression and extensive angiogenesis in breast cancer cell lines [34]. Antioxidant response from junD in Ki-Ras transformed fibroblast cells have been reported to reduce HIF expression and angiogenesis by enhancing PHDs expression [35]. Both antioxidant *N*-acetylcysteine and ascorbate abolish HIF activation through increased hydroxylation [36]. These findings propose the involvement of hypoxia and reactive oxygen species in altering HIF expression through various mechanisms. HIF-1α promoter contains a binding site for necrosis factor-kappa B (NF-ĸB) which conveys up-regulation by oxidative stress [37]. Transcription of HIF-1β is also regulated directly by NF-ĸB [38]. So, it is concluded that PHDs, ARD and VHL all require oxygen to stabilize the HIF-1α structure.

#### pVHL independent pathway

Another regulatory pathway is post-translational modifications of HIF-1 in the presence of oxygen but without the involvement of pVHL protein. Binding of C-TAD and co-activators CBP/p300 are must for transcriptional activation of HIF-1α target genes. In the presence of oxygen, asparagine residue (N^803^) of HIF-1α C-TAD domain gets hydroxylated by factor inhibiting HIF-1 (FIH-1), an asparagine hydroxyl, hinders the interaction between two domains and results in the down-regulation of HIF-1α mediated gene transcription [39–42]. Hypoxic conditions hinder hydroxylation of asparagine and hence establish transactivation of HIF-1α [40–42]. Hence, pVHL dependent pathway stabilizes HIF-1α, while pVHL independent pathway regulates its transactivation.

#### Calcium-mediated regulation

Hypoxia-induced accumulation of calcium also promotes the expression of HIF-1α. Various targets of Ca^2+^/CAM such as CAM kinase II, actin and calcineurin increase the transcriptional expression of HIF-1α. Therefore suppression of Ca^2+^/CAM by CAM-dominant mutants, Ca^2+^ antagonists, i.e. HBC or Ca^2+^ chelators reduces transcriptional activity of HIF and thus angiogenesis [43, 44].

### Oxygen-independent regulation

Despite the role of hydroxylases, there are some other oncogenic regulatory pathways which play an important role in oxygen independent regulation of HIF-1α activity.

#### Growth factor signaling pathway

Various growth factors regulate the expression of HIF-1α through a wide range of signaling cascades, i.e. PI3K/PTEN/AKT or RAS/RAF/MAPK [45]. Most of them regulate translation of HIF-1α while some also regulate transcription. Phosphatidylinositol-4,5-bisphosphate 3-kinase (PI3K) regulates the expression of HIF-1α through its target protein kinase B (AKT) a serine/threonine kinase and further downstream mTOR. mTOR phosphorylates the eukaryotic translation initiation factor 4E (elF-4E) binding protein (4E-BP). 4E-BP disrupts cap-dependent mRNA translation. mTOR dependent phosphorylation of P70S6K (S6K) enhances the activity of its substrate ribosomal protein S6 and promotes HIF-1α translation [46]. The AKT signaling may regulate HIF-1α expression by both mTOR dependent and independent pathways [47]. This pathway is hindered by PTEN, which dephosphorylates PI3K products [47]. Certain growth factors activate RAS which further stimulates MAPK pathway [48]. This pathway involves RAS/RAF/MEK/ERK kinase cascade. An activated extracellular signal-regulated kinase (ERK) phosphorylate MAP kinase-interacting kinase (NNK) and S6K to activate them and 4E-BP1 to inactivate it. MNK phosphorylates elF4E protein to enhance HIF-1α translation [49]. ERK also stimulates transcriptional activation of HIF-1α by phosphorylating CBP/P300 co-activators for enhancing HIF-1α/co-activators complex formation [50].

#### Mdm2 pathway

Mouse double minute 2 homolog (Mdm2) plays an important role along with p53 in cancer. In hypoxic conditions binding of p53 with HIF-1α enhances its degradation through Mdm2 mediated ubiquitination and proteasomal degradation [50]. This degradation occurs in the cytoplasm and controlled by PTEN/PI3K/AKT pathway [51]. Undoubtedly, loss of p53 in the tumor is associated with the enhanced level of HIF-1α expression [52].

#### Through HSP90

Many other minor pathways play a key role in regulating HIF-1α level. Heat shock protein 90 (HSP90) is a molecular chaperone which facilitates synthesis and folding of proteins. Direct interaction of HSP90 with HIF-1α enhances its dimerization with HIF-1β through conformational changes [53]. Geldanamycin, an HSP90 inhibitor enhances HIF-1α degradation even in cells lacking pVHL [54]. Mutations in P^402^/P^534^ sites of HIF-1α do not secure it from Geldanamycin-induced degradation.

## HIF induced metabolic alterations

**Fig. 4:**
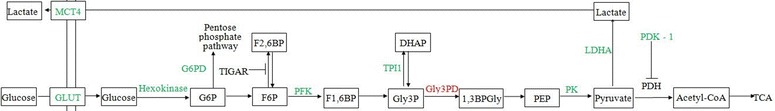
Metabolic targets of HIF-1 in cancer cells. GLUT: glucose transporter; G6P: glucose 6-phosphate; G6PD: glucose 6-phosphate dehydrogenase; F6P: fructose 6-phosphate; F2,6BP: fructose 2,6-bisphosphate; PFK: phosphofructokinase; F1,6BP: fructose 2,6-bisphosphate; Gly3P: glyceraldehyde 3-phosphate; TPI1: triosphosphate isomerase1; DHAP: dihydroxyacetone phosphate; Gly3PD: glyceraldehyde 3-phosphate dehydrogenase; 1,3BPGly: 1,3-bisphosphoglycerate; PEP: phosphoenol pyruvate; PK: pyruvate kinase; LDHA: lactate dehydrogenase; PDH: pyruvate dehydrogenase; PDK1: pyruvate dehydrogenase kinase-1

The functional gain of oncogenes and loss of function of tumor suppressor genes are the main characteristic of cancer cells [55]. This causes uncontrolled proliferation to form a solid mass. So in order to maintain energy level for dividing cells, a continuous supply of anabolic building blocks and energy carriers are established. Alterations of metabolic pathways were added to the six cancer hallmarks by Hanahan and Weinberg [55]. These pathways contain up-regulation of glycolysis, mitochondrial biogenesis, lipid and amino acid metabolism, pentose phosphate pathway and macromolecule biosynthesis. Some of them are shown in Fig. 4.

In comparison with normal cells, cancer cells prioritize lactic acid production (anaerobic glycolysis) instead oxidative phosphorylation even in normoxic conditions also known as Warburg effect [56]. In later studies, it was observed that the dividing lymphocytes also use 90% of their glucose carbon to form lactate, which rules out the probability that anaerobic glycolysis is only associated with cancer cells [57]. HIF-1 and c-Myc are two major regulators of glycolytic enzymes such as hexokinase (HK2), phosphofructokinase (PFK1), triosephosphate isomerase (TPI1) and lactate dehydrogenase (LDHA) [20, 58–60]. At a low level of HIF-1, the end product of glycolysis is converted to acetyl-CoA, which enters into the citric acid cycle. In TCA cycle, high-energy molecules, i.e. FAD and NADH are generated, which further produce a high number of ATPs through electron transport chain in mitochondria. Normal cells prefer anaerobic glycolysis only in the absence of oxygen. In comparison, cancer cells use only anaerobic glycolysis even in abundance of oxygen [58, 61, 62]. As per the research findings, if glucose influx is high enough, only then the percentage of ATP generated from glycolysis goes above that produced from oxidative phosphorylation [63]. Pyruvate kinase an enzyme which converts phosphoenolpyruvate to pyruvate was recently reported as a regulator of Warburg effect [64].

Another glycolytic enzyme responsible for regulatory metabolic pathways at enhanced HIF-1α expression is pyruvate kinase (PK). It encodes by two genes, PKLR and PKM2. PKM2 gene codes for two alternatively spliced transcripts, PKM1 and PKM2. PKM1 is primarily restricted to brain and muscles while PKM2 to fast proliferating cells like cancer [65, 66]. Some glucose depleted cell cultures have been shown to stabilize HIF-1α by inhibiting PHDs through Pyruvate formed from lactate by lactate dehydrogenase [67]. Accumulation of lactate (10 mM) in cancer has been suggested to activate HIF-1 and VEGF [68]. Pyruvate and oxaloacetate inactivate the PHDs, reversal of which requires ascorbate [69]. Thus there are at least two mechanisms to inactivate HIF hydroxylases, one is competition with 2-OG and second by oxidation.

TCA cycle is the second pathway after glycolysis in the metabolism of glucose. Pyruvate, the end product of glycolysis enter TCA cycle only via production of acetyl-CoA. HIF-1α mediated upregulation of pyruvate dehydrogenase kinase reduce acetyl-CoA production through inactivation of pyruvate dehydrogenase enzyme by phosphorylation [70]. Production of NADH by conversion of lactate from pyruvate with the help of lactate dehydrogenase accelerates glycolysis [71]. Another benefit taken by cancer cells from enhanced production of lactate is that they secrete it in tumor microenvironment through MCT4 transporters to other cancer cells, who have not sufficient fuel supply, so to feed them energy.

Pentose phosphate pathway (PPP) is a major pathway for nucleotide biosynthesis through ribose 5-phosphate (R5P) intermediate. p53 plays a major role in controlling both the oxidative and non-oxidative arms of PPP pathway. TP53-induced glycolysis and apoptosis regulator (TIGAR), a target of p53 regulate glycolysis by suppressing the expression of phosphofructokinase-1 and by increasing substrate delivery to citric acid pathway [72]. Cancer cells lacking or mutated p53 lose their control to regulate PPP pathway and hence increases in glycolytic efflux. Glyceraldehyde-3-phosphate dehydrogenase (GAPDH) plays a major role in the regulation of this pathway. Suppressive oxidative phosphorylation reduces NAD^+^/NADH ratio and hence GAPDH activity. Hence down-regulated GAPDH expression creates a boundary for lower glycolytic pathways, creating a high pressure of upper glycolytic substrates, which then enhance reversible non-oxidative PPP pathway for nucleotide biosynthesis [73]. Glucose 6-phosphate dehydrogenase (G6PD) is another important enzyme of PPP pathway which promotes cancer formation by providing NADH [74]. P53 tightly regulate this enzyme by preventing its active dimerization [75]. G6PD is a direct target of HIF-1 to transactivate it [76]. Surprisingly, G6PD and VEGF crosstalk in cancer cells and are highly associated with angiogenesis [77, 78].

Catabolism of glutamine is another key metabolic pathway for cancer cells. End products of glutaminolysis serve as intermediates for TCA cycle. Like glycolysis, glutaminolysis provides not only ATP but also some crucial precursors for cell proliferation [79–81]. C-Myc transactivates alanine–serine–cysteine (ASCT2) and system N (SN2) transporters for glutamine and up-regulates glutaminase (GLS1) expression [82]. The high rate of lactate and alanine excretion per mole of glutamine, like the evident insufficiency of Warburg effect, has been seen in proliferating cells [78, 83]. Rather than regular discernment that normal cells use glutamine as a nitrogen source, glutamine digestion in tumor cells bring high intracellular nitrogen that must be discharged as alanine or ammonia. Glutaminase enzyme removes its amido group as ammonia. In glioblastoma cells, a large part of glutamine’s amino groups was likewise lost in α-ketoglutarate (α-KG) producing reactions (glutamate dehydrogenase and alanine aminotransferase) [84]. In this way, usage of glutamine as an anaplerotic precursor and source of NADPH results in the discharge of large fraction of glutamine-derived carbon and nitrogen. A portion of secreted molecules may, therefore, be utilized as precursors for hepatic gluconeogenesis, hence giving more fuel for tumor digestion. At first look, these seem, by all accounts, to be side effects of metabolic inefficiency. However, they may actually give a logical and specialized metabolism that empower cell growth and metabolism. In this way, HIF-1α alter a number of metabolic pathways that play their role in cancer by providing energy to the cell for proliferation, growth, and survival. Targeting specific substrates of these pathways may help to control cancer progression.

## Conclusions

Both HIF-1 and metabolic pathways participate in cancer progression by giving an enhanced supply of energy (metabolic pathways) and sufficient oxygen (HIF-1). A sequence of signaling cascades is involved by these two factors to survive cancer cells through the harsh environment and immune surveillance. Both the pathways are up-regulated in cancer. Warburg effect, which is a characteristic of altered glycolytic pathways in cancer, is also seen in dividing lymphocytes and embryonic stem cells. HIF-1, a mark of the hypoxic condition is also seemed to be regulated in an oxygen-independent manner. This complex regulation contributes to the propagation of cancer through multiple pathways.

### Future perspectives

The involvement of HIF in cancer progression directs our intention to discover anticancer drugs which inhibit HIF-1 expression directly or indirectly by regulating HIF-1α mRNA, HIF-1 α protein translation, HIF-1α protein degradation, HIF-1α and HIF-1β subunit interactions and HIF-1α DNA binding activity. Numerous studies focused on cancer, entail discovery of drugs which are synthetic in nature. A number of studies are reported to compile mode of action of these anticancer drugs in contrast with the HIF-1 regulation [20, 85–91]. However, even after extensive research, there is a gap to target cancer with a single molecule because of multiple survival pathways, different infected organs and huge side effects of synthetic drugs. From last decade, researchers have identified some natural plant compounds, i.e. vinca alkaloids, taxanes, camptothecins [92] which have fewer side effects than synthetic ones. Recently, a new natural compound sulforaphane was identified, which eliminates cancer stem cells in many cancer types [93–95]. Multiple survival pathways make trouble for anticancer drug discovery. Some substrates which are common for multiple responsible pathways for cancer may help to target it with a novel universal drug in future. This requires extensive study of signaling cascades and its regulation on each step.

## Abbreviations

HIF-1: hypoxia-inducible factor
ROS: reactive oxygen species
ARNT: aryl hydrocarbon receptor nuclear translocator
AhR: aryl hydrocarbon receptor
bHLH: basic helix loop helix
PAS: Per ARNT Sim
ODD: oxygen-dependent domain
N-TAD: N-terminal transactivational domain
C-TAD: C-terminal transactivational domain
CBP: C-TAD binding protein
KAT: lycin acetyl transferase
DNA: deoxyribonucleic acid
VEGF: vascular endothelial growth factor
EGF: epidermal growth factor
TNF-α: tumor necrosis factor-α
GLUT1: glucose transporter 1
SGLT: sodium–glucose transporter
ATP: adenosine triphosphate
MCT-4: monocarboxylate transporter-4
CA: carbonic anhydrase
VHL: von Hippel-Lindau
PHDs: prolyl-4-hydroxylases
HPH: HIF-1 prolyl hydroxylase
2-OG: 2-oxoglutarate
ARD-1: arrest defective-1
shRNA: small hairpin ribonucleic acid
NF-ĸB: necrosis factor-kappa B
FIH-1: factor inhibiting HIF-1
PI3K: phosphatidyl inositol-4,5-bisphosphate 3-kinase
m-TOR: mammalian target of rapamycin
elF-4E: eukaryotic translation initiation factor-4E
4EBP: elF-4E binding protein
PTEN: phosphatase and tensin homolog
MAPK: mitogen-activated protein kinase
ERK: extracellular signal-regulated kinase
MNK: MAP kinase-interacting kinase
Mdm2: mouse double minute 2 homolog
HSP90: heat shock protein 90
CAM: calmodulin-dependent kinase
HK2: hexokinase 2
PFK1: phosphofructokinase 1
TPI1: triphosphate isomerase 1
LDHA: lactate dehydrogenase
TCA: tricarboxylic acid
FAD: flavin adenine dinucleotide
NADH: nicotinamide adenine dinucleotide
PPP: pentose phosphate pathway
R5P: ribose 5-phosphate
TIGAR: TP-53-induced glycolysis and apoptosis regulator
GAPD: glyceraldehyde-phosphate dehydrogenase
G6PD: glucose 6-phosphate dehydrogenase
PK: pyruvate kinase
ASCT: alanine, serine, cysteine transporter 2
SN2: system n-2 transporter
GLS1: glutaminase 1
αKG: α-ketoglutarate
mRNA: messenger ribonucleic acid
